# Unveiling the impact of varied load intensities on para-biathletes’ performance in rifle shooting

**DOI:** 10.3389/fpsyg.2026.1732344

**Published:** 2026-07-08

**Authors:** Runji Wang, Qianlan Zhang, Ye Liu, An Wen

**Affiliations:** 1 School of Physical Education and Health, Changchun Normal University, Changchun, Jilin, China; 2 Department of Scientific Research, Changchun Normal University, Changchun, Jilin, China; 3 School of Science, Xi’an Technological University, Xi’an, Shanxi, China

**Keywords:** hit rate, load intensities, para-biathlon, prone shooting, rifle shooting

## Abstract

The study investigated the effect of varied load intensities on shooting performance after standing and seated gliding by simulated Paralympics in conditions. Sixteen well-trained Chinese biathletes (average age 20.52 ± 0.88 years) completed tests at different transition times (100% HRmax, maximum heart rate; 85% HRmax, and 70% HRmax) in the sitting and standing postures. Subjects are required to glide in a double-braced cane maneuver and then complete the shot in a prone shooting position after gliding. Physiological measurements (HR, blood lactate concentration, and rating of perceived exertion) and shooting performance (hit number, shooting time, range time) were collected. For standing sliding, the mean number of hits of 70% HRmax was significantly different compared to 100% HRmax and 85% HRmax. For seated sliding, the shooting performance results revealed in the 2nd transition round, 85% HRmax had significantly higher shot hits than 100% HRmax. The load intensity of 85% HRmax has the best effect on para-biathlon athletes. Under this intensity, athletes have the shortest shooting time, the largest number of total shooting hits, and the highest hit rate and transition efficiency.

## Introduction

Para-biathlon consists of cross-country skiing and shooting, in which athletes complete the competition in a sitting (LW12) or standing (LW8) position, enter the shooting area after gliding at high speed, and complete five consecutive prone shots on the shooting line, with 2–4 transitions between the two sub-events (depending on the competition distance), completing a total of 10 or 20 shots (Heinrich et al., 2020; Gallicchio et al., 2019; Jonsson Karstrom et al., 2019; Luchsinger et al., 2019a). Previous studies have emphasized the significance of fast skiing and accurate shooting for success in winter events (Heinrich et al., 2020). With athletes continuously enhancing their competitive abilities, shooting performance has become a pivotal factor in determining success at international competitions (Ohtonen et al., 2020; Pelin and Mereuta, 2018; Köykkä et al., 2020; Laaksonen et al., 2020). In short-distance competitions, shooting performance accounts for about 35% of the overall performance, and in long-distance competitions or pursuit races (starting one by one), it reaches or even exceeds 50% of the overall performance due to time penalties (Laaksonen et al., 2018a,b; Luchsinger et al., 2020). Hence, shooting performance is increasingly crucial for the overall performance in para-biathlon competitions (Maier et al., 2019).

The transition from cross-country skiing to shooting places significant demands on the athletes’ physiological functions, mental focus, and movement stability (Luchsinger et al., 2020). Currently, the issue of shooting performance has garnered increasing attention, particularly as the gap in gliding ability among Para-biathletes gradually narrows (Benum et al., 2021). On the one hand, athletes in para-biathlon competitions use standing or sitting postures, which affect shooting performance through the control of body posture and stability maintenance during gliding and prone shooting; On the other hand, there is currently limited research on prone shooting posture compared to standing shooting. Given that movement intensity has less impact on prone shooting performance, stability in shooting performance holds greater significance for overall athlete performance (Ihalainen et al., 2018). On biathlon transitions, the crucial factor for shooting stability lies in the athlete’s ability to restore their heart rate (HR) before reaching the firing line (Heinrich et al., 2021). In essence, the pre-shooting HR plays a vital role in determining shooting performance (Sattlecker et al., 2017; Vickers and Williams, 2007). Sattlecker et al. (2017) analyzed the speed and HR of winter athletes during their approach to the firing line. The study identified three different types of entries into the shooting area full-speed entry, appropriate deceleration entry, and active deceleration entry. Each entry type corresponded to specific HR intervals of 100% HRmax, 85% HRmax, and 70% HRmax, respectively. The subject’s maximum heart rate was 200 beats/min, and the athlete used 100% maximum heart rate, i.e., reached a heart rate of 200 beats/min to start gliding and held it for a period of time. Shooting performance can be primarily divided into two parts: shooting hit rate and shooting time, which depend on the ability of aerobic energy supply, mental quality under load, and control of body balance (Köykkä et al., 2020; Laaksonen et al., 2020). Previous studies have demonstrated that adequate aerobic energy supply contributes to stable physical condition, mental focus, and sustained precision performance in shooting events (Tønnessen et al., 2015). Pre-shooting HR is mainly affected by the intensity exercise prior the firing area as respiration and other physiological parameters. However, the impact of pre-shooting HR on shooting performance in a fatigued state has not been extensively studied, particularly when simulating disabled winter athletes gliding to shooting, entering the shooting area, and undergoing a series of shooting processes under strict laboratory conditions (Ortega and Wang, 2018). In summary, shooting performance plays a crucial role in biathlon, particularly after transitioning from skating to shooting. Pre-shooting HR is mainly influence by the intensity exercise prior the firing area as respiration and other physiological and psychological parameters. Therefore, this study aimed to examine the influence of pre-shooting HR on shooting performance following seated gliding. The study simulated the Paralympics under controlled laboratory conditions, to provide theoretical support and methodological recommendations for speed regulation strategies and HR control when athletes transition from gliding to shooting in the Paralympic Games.

## Materials and methods

### Subjects

Sixteen well-trained Chinese biathletes (Chinese national para-biathlon team), volunteered for this study. All athletes were registered with the official Paralympic classification system for para biathlon. All participants were classified as LW8 and LW12 class athletes, which is the eligible functional class for para-biathlon participation. This classification was determined by official international classification panels based on their physical function, mobility, and visual ability, which is closely related to postural stability and gliding performance during skiing and shooting.

**FIGURE 1:**
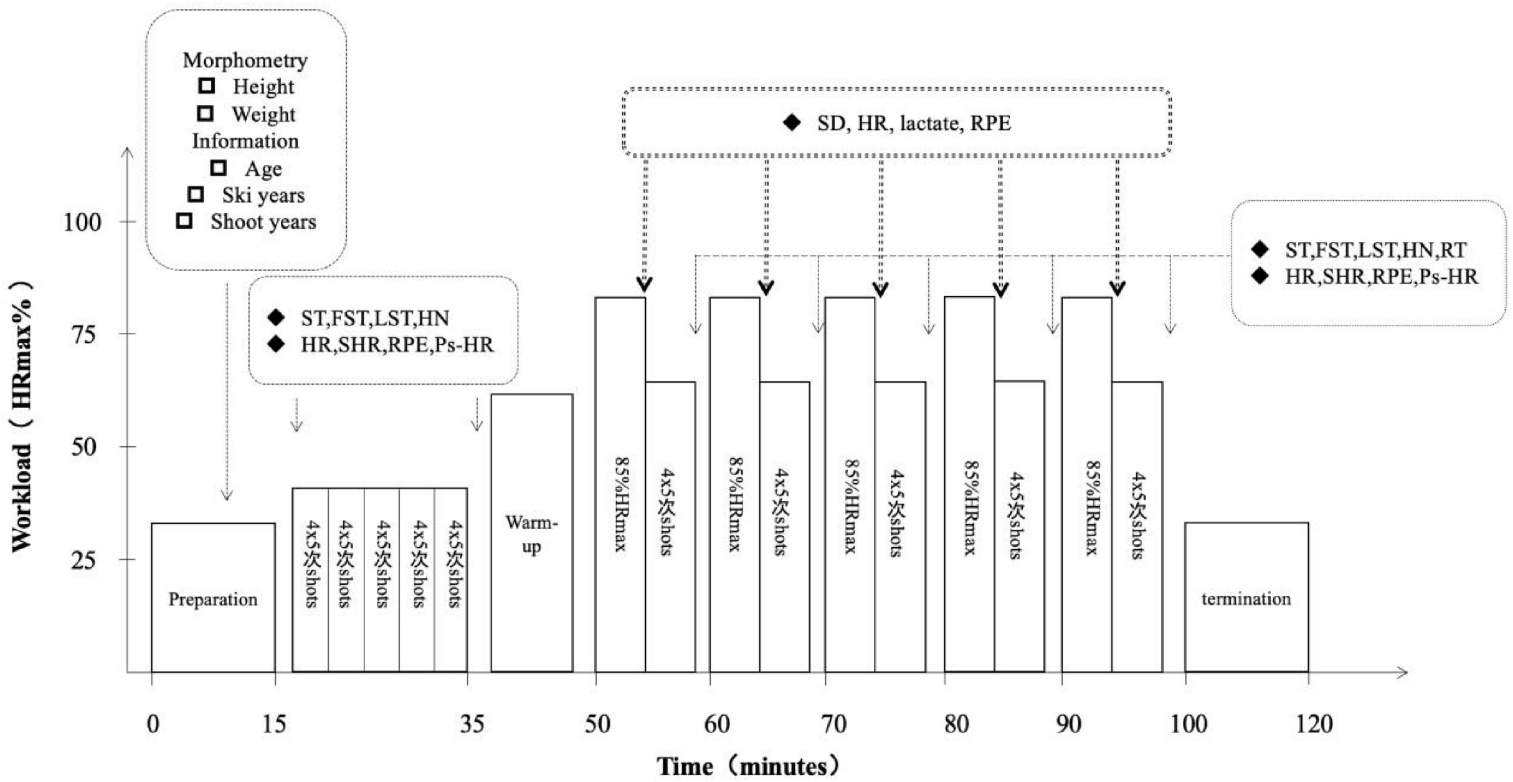
Testing procedures. ST, shooting time; HN, hit number; HR, heart rate; Ps-HR, pre-shooting heart rate; BLA, blood lactate concentration; CT, core temperature; GS, grip strength; RPE, rating of perceived exertion; SD, skiing distance; RT, range time; FST, first shooting time; LST, last shooting time.

They were familiar with treadmill roller skiing due to regular testing and training in the laboratory. Sample size was calculated *a priori* using G*Power 3.1.9.7 software. For a repeated-measures ANOVA with four groups and two measurements, parameters were set at α = 0.05, power = 0.80, and effect size = 0.25, yielding a minimum of 12 participants per group. To account for potential sample attrition, 16 participants per group were included in the present study. The 16 biathletes participating in the study had a mean age of 20.52 years, a mean body height of 1.78 m, a mean body mass of 70.98 kg, a mean body mass index (BMI) of 22.32 kg/m^2^, a mean skiing experience of 7.82 years, and a mean shooting experience of 2.13 years. A 1-week washout period was implemented between experimental trials to minimize residual effects and ensure full recovery between conditions. All subjects refrained from high-intensity exercise for 3 days before each experiment. In addition, the subjects maintained regular daily habits and did not consume tea, functional drinks, or nutritional supplements during the experiment. Informed consent was obtained from all subjects. The institutional ethical committee of the Capital University of Physical Education and Sports, Beijing, China approved all procedures and protocols (No. 2020A81). The study followed in accordance with the Declaration of Helsinki. An overall experiment timeline is shown in Figure 1.

### Exercise protocol

Following the commonly used block experimental design in biathlon research abroad, the subjects in this study were tested using a traditional double pooling ergometer (DPE) on the Thorax Trainer A/S (Kokkedal, Denmark). The Thorax Trainer is an integrated training ski machine that utilizes an advanced non-elastic rope drive system (Sattlecker et al., 2015; Gallicchio et al., 2016; Luchsinger et al., 2016; Panissa et al., 2016; Rosso et al., 2017).

The study used a repeated-measures own-control experimental design. Considering the feasibility of the experimental procedure and the acceptability of each subject, subjects entered the laboratory for the first time to perform the basal value test and to familiarize themselves with the test procedure and the experimental process (Sattlecker et al., 2017). The subjects underwent a total of six experiments each, consisting of three tests in both sitting and standing postures, with different transition times (100% HRmax, 85% HRmax, and 70% HRmax) in Figure 1. To mitigate practice and fatigue effects, the sequence of the tests was offset, and there was a 1-week interval between the two experiments. The tests were conducted at the same time of day (within ±1 h) to minimize any time-related interference with the test results. HRmax refers to the maximum heart rate. In this study, “HRmax 100%” is defined as the exercise intensity ranging from 85% to 100% of HRmax.

Preparation (0–15 min): Participants first completed a morphometric assessment, including measurements of height, weight, age, and years of skiing and shooting experience. Baseline measurements of standing heart rate (HR), standing heart rate recovery (SHR), rate of perceived exertion (RPE), and perceived stress HR (Ps-HR) were also collected.

Pre-test familiarization (15–35 min): This phase consisted of four consecutive blocks of familiarization trials. Each block involved skiing at a submaximal intensity followed by a shooting sequence (4 × 5 shots). During each block, measurements of skiing time (ST), first shot time (FST), last shot time (LST), and shooting accuracy (HN) were recorded, alongside HR, SHR, RPE, and Ps-HR.

Warm-up (35–50 min): A standardized 15-min warm-up was performed to prepare the cardiovascular and musculoskeletal systems for the subsequent high-intensity exercise.

Main experimental trials (50–100 min): The main protocol consisted of four identical experimental blocks. Each block comprised two consecutive phases:

Skiing phase: Participants skied at an intensity corresponding to 85% of their maximum heart rate (HRmax).

Shooting phase: Immediately after skiing, participants completed a shooting sequence of 4 × 5 shots. During each block, skiing distance (SD), HR, blood lactate concentration, and RPE were measured. Additionally, ST, FST, LST, HN, RT, HR, SHR, RPE, and Ps-HR were recorded during the shooting phase.

Termination (100–120 min): Following the final experimental block, participants entered a 20-min cool-down and recovery period to allow physiological parameters to return to baseline levels.

### Physiological indicators measurement

#### Heart rate

Subjects entered the laboratory wearing a heart rate monitor (Polar H10, Finland) and sat still for 10 min to obtain a basal heart rate. Then, after 15 min of warm-up exercise, subjects were ready to start the test. Heart rate was monitored during the completion of a predetermined exercise program, with heart rate measured before and after shooting without load, and before and after each set of shots with exercise load until all shots were completed.

#### Pre-shooting heart rate

Pre-shooting heart rate was the heart rate of the subject at the 1st shot of each firing under exercise load.

#### Blood lactate concentration

Lactate was obtained by testing ear blood with a blood lactate meter (Lactate Scout, Germany). A calibration test was performed to ensure the reliability of the results of the device, and the subjects were allowed to sit in a quiet state for 10 min to obtain the basal value of blood lactate, and then blood samples were collected from the subjects’ earlobe capillaries immediately after each glide to determine the concentration of blood lactate, and the results were recorded.

### Subjective indicator measurements

#### Rating of perceived exertion

The 6–20 Borg rating of perceived exertion (RPE) scale was used to assess subjective exertion throughout the protocol. Prior to data collection, all athletes received standardized instructions on the use of the scale, with clear definitions of the range (6, no exertion at all; 20, maximal exertion) and examples of corresponding exercise intensities. Athletes were familiarized with the scale during a pre-test familiarization session to ensure consistent interpretation. RPE ratings were collected verbally by a trained researcher immediately after each skiing block and shooting sequence, both under loaded conditions (during the main trials) and in the unloaded pre- and post-shooting assessments.

### Shooting performance measurement

#### Shooting time

The time taken by the subject to hold the gun from ready to fire to fire each time during the test. A double-row 10-channel electronic timer (China) was used to record the firing time indicator.

#### First shooting time

Time taken by the subject from arrival at the firing mat to the first shot fired. A double-row 10-channel electronic timer (China) was used to record the firing time indicator.

#### Last shooting time

Time taken by the subject from firing the last round shot to completing the five shots. A double-row 10-channel electronic timer (China) was used to record the firing time indicator.

#### Range time

The time taken by the subject to enter the firing area and to leave the firing area to reconvert for gliding.

#### Hit number

The number of hits for the subject’s five shots after each glide. The shooting targets were standard biathlon competition targets: 45 mm in diameter for prone shooting, consistent with official International Biathlon Union (IBU) regulations.

#### Efficiency performance

Efficiency Performance (EP) was calculated as the ratio of total shooting hits to total shooting time, using the formula:


E⁢P⁢T⁢o⁢t⁢a⁢l⁢S⁢h⁢o⁢o⁢t⁢i⁢n⁢g⁢T⁢i⁢m⁢e/T⁢o⁢t⁢a⁢l⁢N⁢u⁢m⁢b⁢e⁢r⁢o⁢f⁢H⁢i⁢t⁢s

This metric was chosen to quantify shooting efficiency by integrating both accuracy (hits) and speed (time) into a single composite score, which is critical for evaluating performance in biathlon, where rapid, accurate shooting directly impacts race outcomes. While not yet widely adopted in biathlon research, this ratio-based approach aligns with established efficiency models in other precision sports (e.g., archery, pistol shooting) and provides a more holistic assessment of shooting performance than either accuracy or time alone. This choice was motivated by the need to capture the trade-off between speed and accuracy, a key constraint in para-biathlon competition, where athletes must balance postural stability, mobility limitations, and shooting precision.

### Experimental environment control and site layout

**FIGURE 2:**
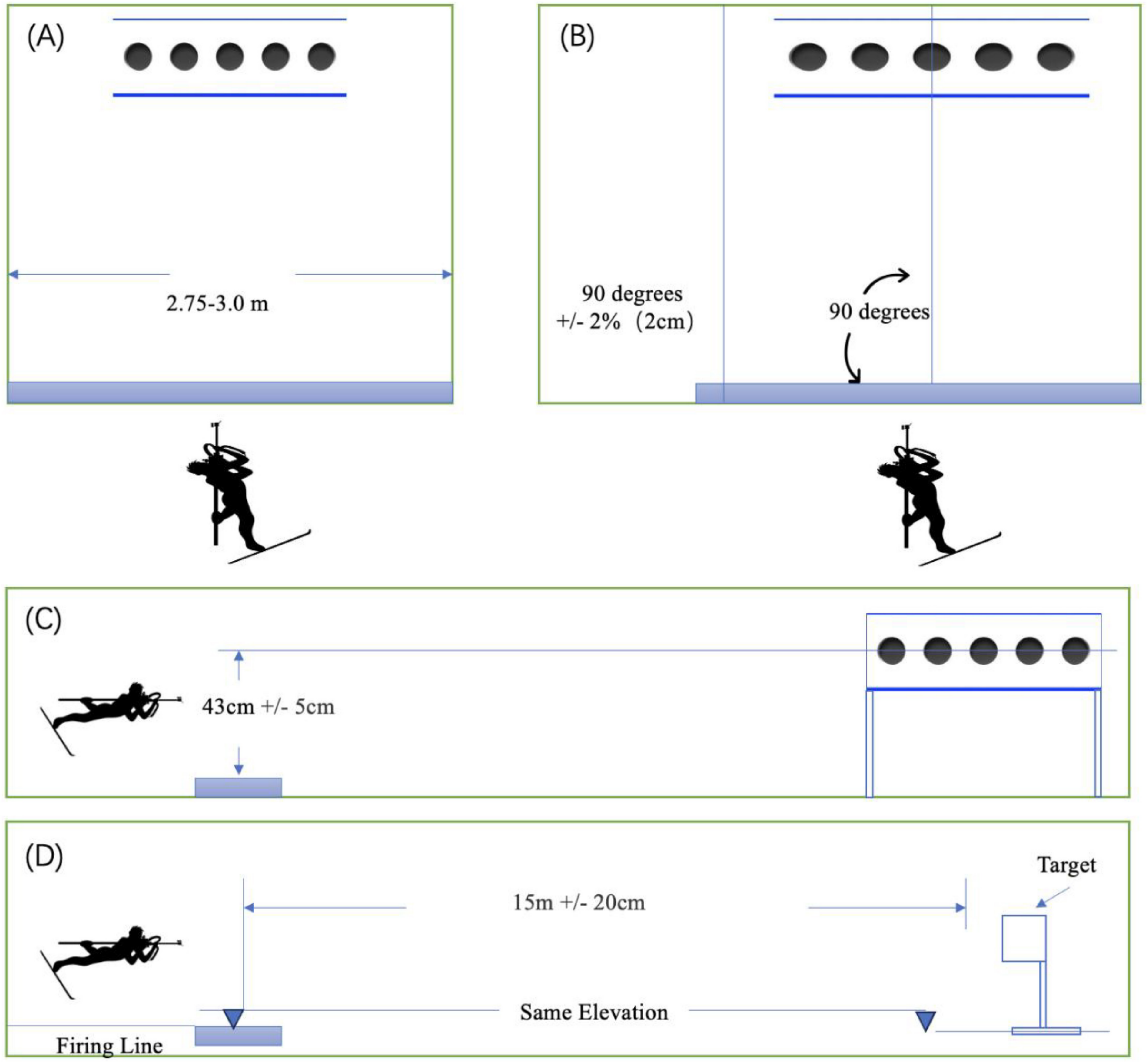
Shooting line width, target location, target height and shooting distance. **(A,B)** Width and angle of firing line. **(C)** Height of target location. **(D)** Distance from firing line to target location.

The campaign environmental conditions were set based on the experimental purpose and protocol. The temperature was controlled between 21 °C and 25 °C, while the humidity was maintained at approximately 45% (Hakkarainen et al., 2016). Wind conditions were regulated using a tower fan (Model ZAC10B; MEDIA, CHINA) with a wind speed of 2–3 m.s^–1^. The ski machine and shooting area were positioned 3 m apart. The shooting targets were designed according to the guidelines provided by World Para Nordic Skiing in their “2021/2022 Rules and Regulations,” the Biathlon Operations Manual, and the Biathlon Range and Equipment Certification Manual issued on 5 October 2021. The targets were set with specific size, shape, height, and distance requirements. The background behind the target was white, extending from the ground to 1 m above the top edge of the target. The rifle used in the study had a total length of 850 mm, a trigger weight of 500 g, a caliber of 4.55 mm, and a weight of 5 kg. The firing line width, target position, target height, and firing distance are illustrated in Figure 2.

The subjects were actively encouraged to give their best effort during the test to enhance the accuracy of the test results (Stöggl et al., 2016). In terms of clothing, subjects were asked to wear the same clothing all three times (Skiing, 2019). During the simulated seated glide, the seat height was adjusted between the hip and seat contact point and the ski machine equipment was 40 cm, and the athlete remained in contact with the seat.

### Statistical analyses

All statistical analyses were performed using Microsoft Office Excel 2013 and SPSS 22.0 software, and graphs were constructed using GraphPad Prism 5. Data are presented as the mean ± standard deviation (M ± SD). Normality tests were performed for all variables. For non-normally distributed data, the Friedman non-parametric test was used to analyze differences between resting and loading conditions. A repeated-measures analysis of variance (ANOVAs) was applied to examine changes in kinematic, physiological, psychological, and mechanical variables across different transition rounds. The Kruskal-Wallis non-parametric test was used for shooting time and shooting accuracy; when a significant main effect was detected, *post hoc* pairwise comparisons were performed using the Dwass-Steel-Critchlow-Flinger test. Canonical correlation analysis was used to evaluate relationships among kinematic, physiological, psychological, and mechanical indicators across transition rounds. Spearman’s correlation analysis was performed to examine associations of shooting time and accuracy with transition round ranking and total ranking. Independent-samples *t*-tests were used for normally distributed data to assess the effects of gliding posture and transition duration on psychophysiological responses and shooting stability. Non-normally distributed data were analyzed using the Friedman non-parametric test. The significance level was set at α ≤ 0.05, with *p* < 0.05 considered statistically significant and *p* < 0.01 considered highly significant.

## Results

### Shooting performance measurement

#### Hit number

**FIGURE 3:**
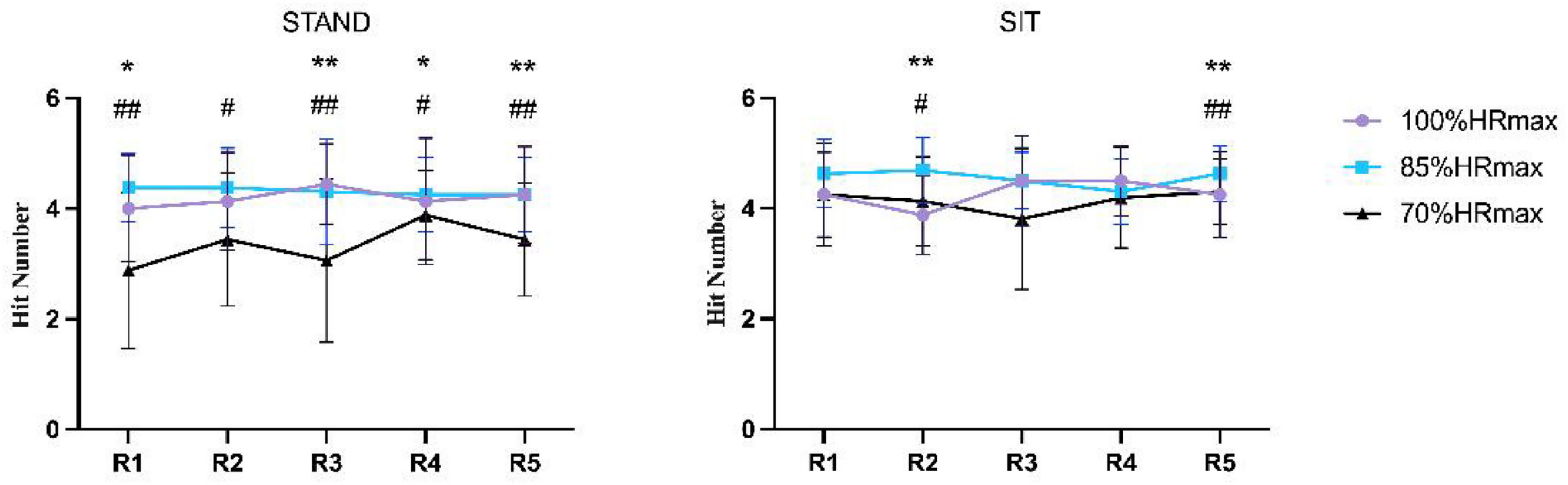
Mean number of hits (HN) during standing (left) and seated (right) shooting at different intensities (100% HRmax, 85% HRmax, and 70% HRmax) across five transition rounds (R1nding *P < 0.05, **p < 0.01, significant difference compared with 100% HRmax. ^#^*P* < 0.05, ^##^*p* < 0.01, significant difference compared with 85% HRmax. HN, number of hits. Error bars represent standard error of the mean (SEM).

For the standing condition: As shown in Figure 3 (left), no significant difference in mean hits was observed between 100% HRmax and 85% HRmax (*p* = 0.03). The 70% HRmax condition yielded significantly fewer hits than both 100% HRmax (*p* = 0.02) and 85% HRmax (*p* = 0.01). In the 2nd transition round, 85% HRmax had more hits than 100% HRmax (*p* = 0.02), while 70% HRmax had fewer hits than 85% HRmax (*p* = 0.03).

For the seated condition: As shown in Figure 3 (right), the pattern was similar to standing: no significant difference between 100% HRmax and 85%HRmax, with 70% HRmax showing fewer hits than both. In the 2nd transition round, 85% HRmax had more hits than 100% HRmax, and 70% HRmax had fewer hits than 85% HRmax.

#### First shooting time

**FIGURE 4:**
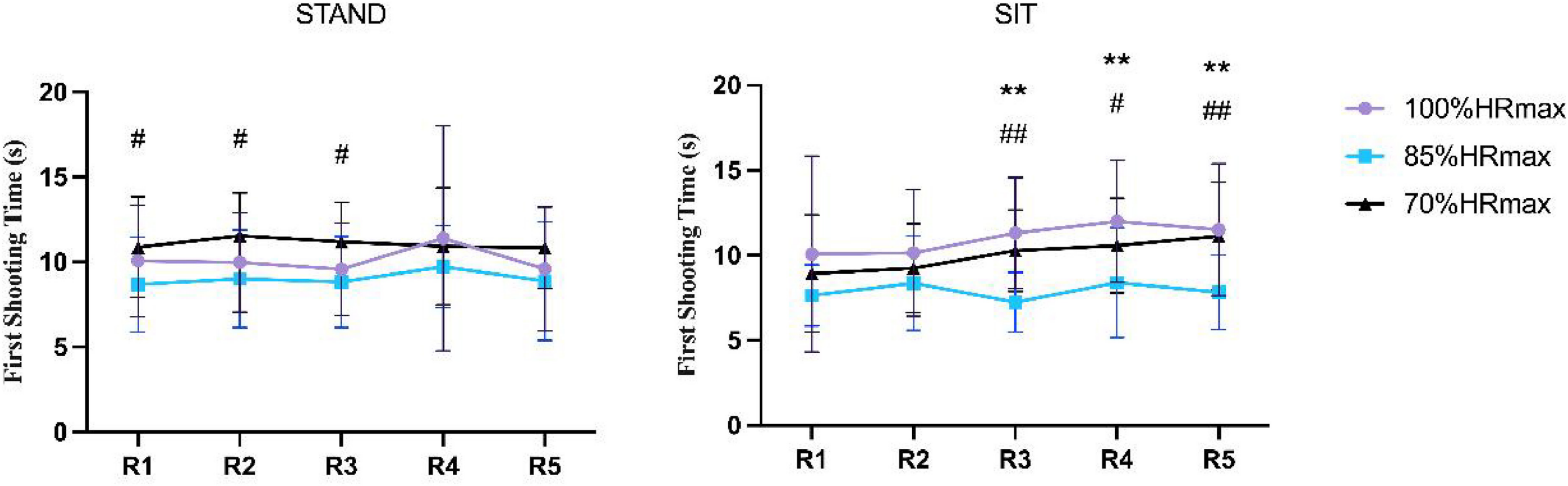
Effect of varied load intensities on first shot time. **Significant difference compared with 100% HRmax. ^#^*P* < 0.05; ^##^*P* < 0.01 Significant difference compared with 85% HRmax condition; ST, shooting time.

As shown in Figure 4, at transition rounds 1 (F = 3.147, *t* = 4.385, *p* = 0.03), 2 (F = 1.081, *t* = 2.991, *p* = 0.02), and 3 (F = 8.023, *t* = 3.291, *p* = 0.03), first shot time was significantly lower for 85% HRmax than for 100% HRmax, and first shot time for 70% HRmax was not significantly different from 100% HRmax (*p* = 0.06). In the R3 (F = 4.184, *t* = −4.094, *p* = 0.03), R4 (F = 0.014, *t* = −2.053, *p* = 0.02), and R5 (F = 2.352, *t* = −3.421, *p* = 0.04) conversion rounds, 70% HRmax’s first shot time was significantly higher than 85% HRmax.

#### Last shooting time

**FIGURE 5:**
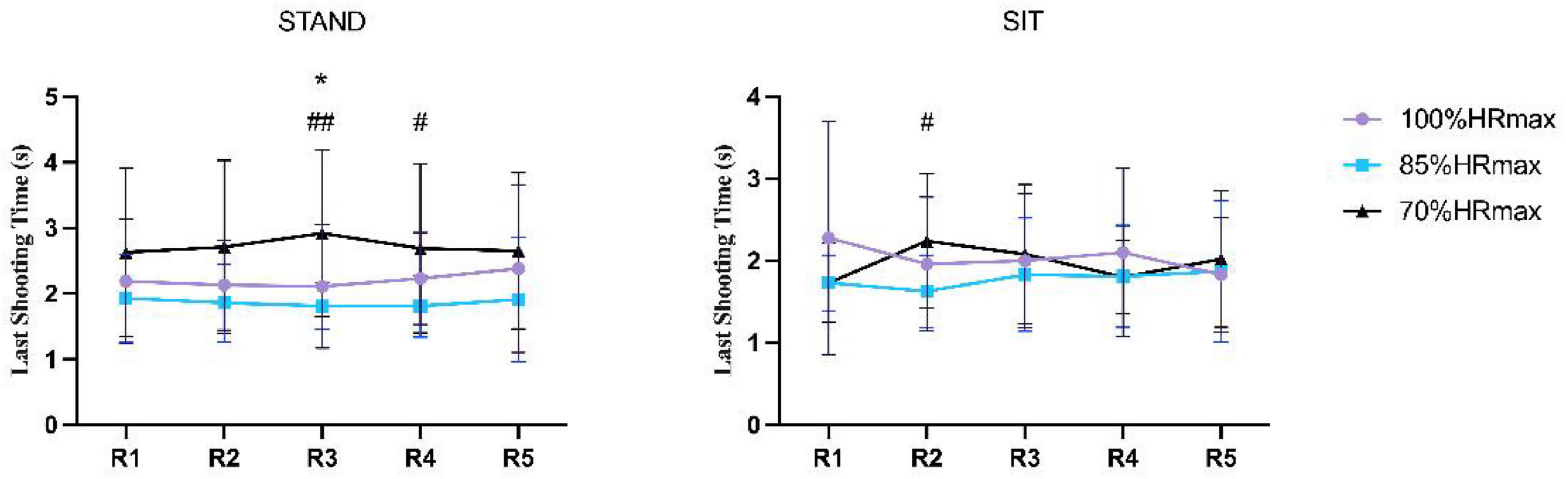
Effect of varied load intensities on the last shooting time. *P < 0.05, **P < 0.01 Indicates a significant difference compared to 100% HRmax. ^#^*P* < 0.05, ^##^*P* < 0.01 Indicates a significant difference compared to 85% HRmax. ST_5, time of the last shot.

As shown in Figure 5, for standing sliding, in the 3rd and 4th transition round, 85% HRmax and 100% HRmax had significantly lower last shooting time than 70% HRmax (F = 16.112, *t* = −1.319, *p* = 0.03). For seated sliding, in the 2nd transition round, 70% HRmax had a significantly higher last shooting time than 85% HRmax (F = 11.016, *t* = −2.604, *p* = 0.04).

#### Efficiency performance

**FIGURE 6:**
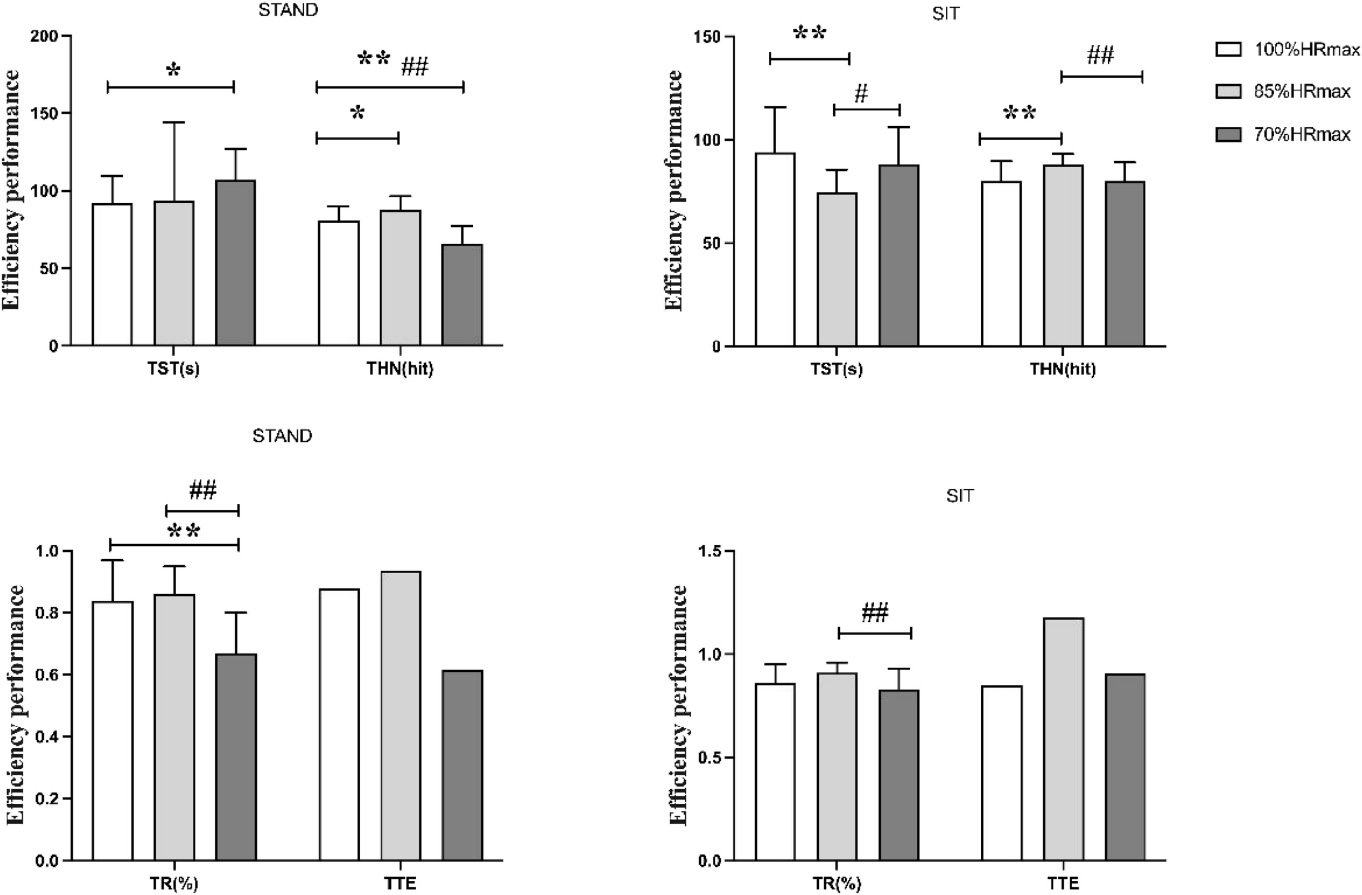
Effect of varied load intensities on efficiency performance. *P < 0.05, **P < 0.01 Indicates a significant difference compared to 100% HRmax. ^#^*P* < 0.05, ^##^*P* < 0.01 Indicates a significant difference compared to 85% HRmax. TST, total shot time; THN, the total number of hits; TR, total hit efficiency; TTE, total transition efficiency.

As shown in Figure 6, in terms of total number of hits, 85% HRmax was significantly higher than 100% HRmax (F = 3.220, *t* = −2.893, *p* = 0.007), and the total number of hits for 70% HRmax was also significantly lower than 85% HRmax (F = 2.359, *t* = 3.020, *p* = 0.008); in terms of total hit rate, 70% HRmax was significantly lower than 85% HRmax (F = 4.116, *t* = 2.819, *p* = 0.006). In addition, according to the total transition efficiency score = the total number of hits (THN)/total firing time (TST), the score of the 100% HRmax group was 0.851 (95% CI: 0.72–0.98), the score of the 85% HRmax group was 1.176 (95% CI: 1.02–1.33), and the score of the 70% HRmax group was 0.906 (95% CI: 0.81–1.00), which shows that from the total conversion efficiency score, the 85% HRmax group > 70% HRmax group > 100% HRmax group. This significant effect was observed in both standing and seated postures.

### Physiological indicators measurement

#### Pre-shooting HR

**FIGURE 7:**
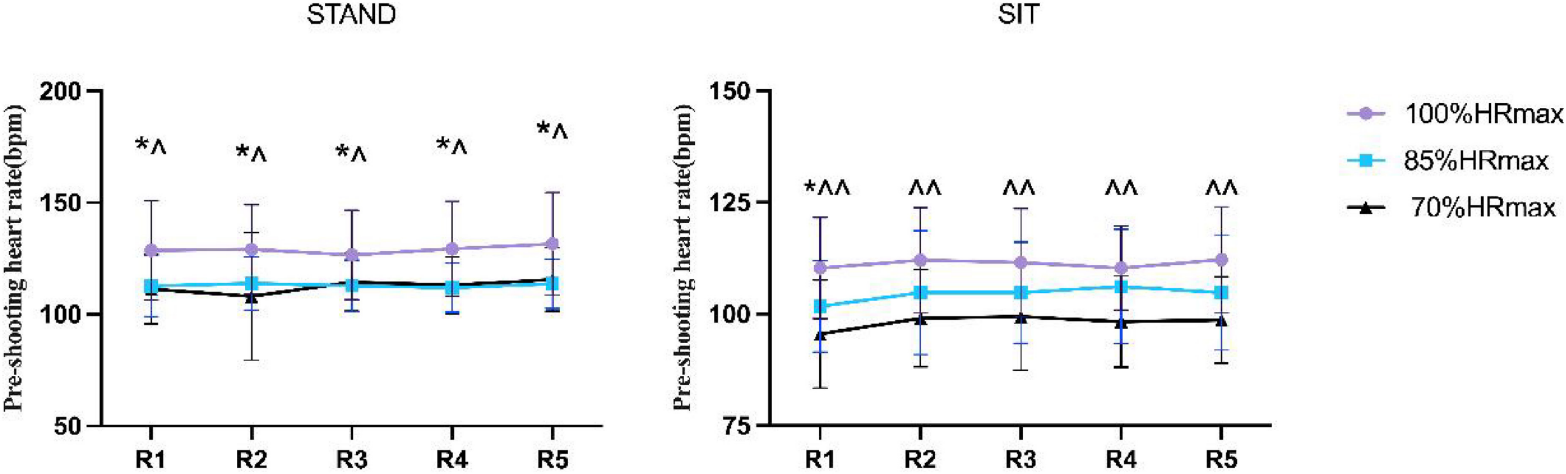
Effect of varied load intensities on HR before shooting. *Significant difference 85% HRmax compared to 100% HRmax. ^∧^*P* < 0.05, ^∧∧^*P* < 0.01 Significant difference 70% HRmax compared to 100% HRmax.

As shown in Figure 7, shooting line HR was significantly lower in rounds 1 (F = 1.451, *t* = 3.145, *p* < 0.01), 2 (F = 4.260, *t* = 4.594, *p* = 0.003), 3 (F = 1.709, *t* = 3.935, *p* = 0.000), 4 (F = 2.060, *t* = 4.176, *p* < 0.01), and 5 (F = 0.844, *t* = 4.215, *p* = 0.005) conversion rounds, 85% HRmax was significantly lower than 100% HRmax; similarly, 70% HRmax was significantly lower compared to 100% HRmax in rounds 1 (F = 1.265, *t* = 3.977, *p* = 0.004), 2 (F = 5.076, *t* = 5.394, *p* = 0.004), 3 (F = 3.456, *t* = 4.362, *p* = 0.002), 4 (F = 4.028, *t* = 4.791, *p* = 0.005), and 5 (F = 1.765, *t* = 4.951, *p* = 0.003) conversion rounds were also significantly different. However, no significant difference was seen between 70% HRmax and 100% HRmax (*p* = 0.07).

**FIGURE 8:**
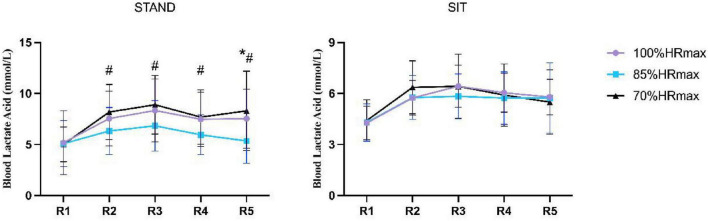
Effect of varied load intensities on blood lactate concentration. *Significant difference compared to 100% HRmax. ^#^Significant difference compared to 85% HRmax.

As shown in Figure 8, blood lactate concentration was significantly lower in 70% HRmax than 100% HRmax in the R5 (F = 1.877, *t* = 2.415, *p* = 0.04) conversion round; no significant difference was seen between 70% HRmax and 100% HRmax (*p* = 0.06). However, 70% HRmax was significantly lower compared to 100% HRmax in rounds 2 (F = 0.184, *t* = −2.102, *p* = 0.03), 3 (F = 0.358, *t* = −2.172, *P* < 0.05), 4 (F = 1.284, *t* = −2.087, *p* = 0.04), 5 (F = 2.755, *t* = −2.638, *p* = 0.023) also had significant differences in conversion rounds.

#### Rating of perceived exertion

**FIGURE 9:**
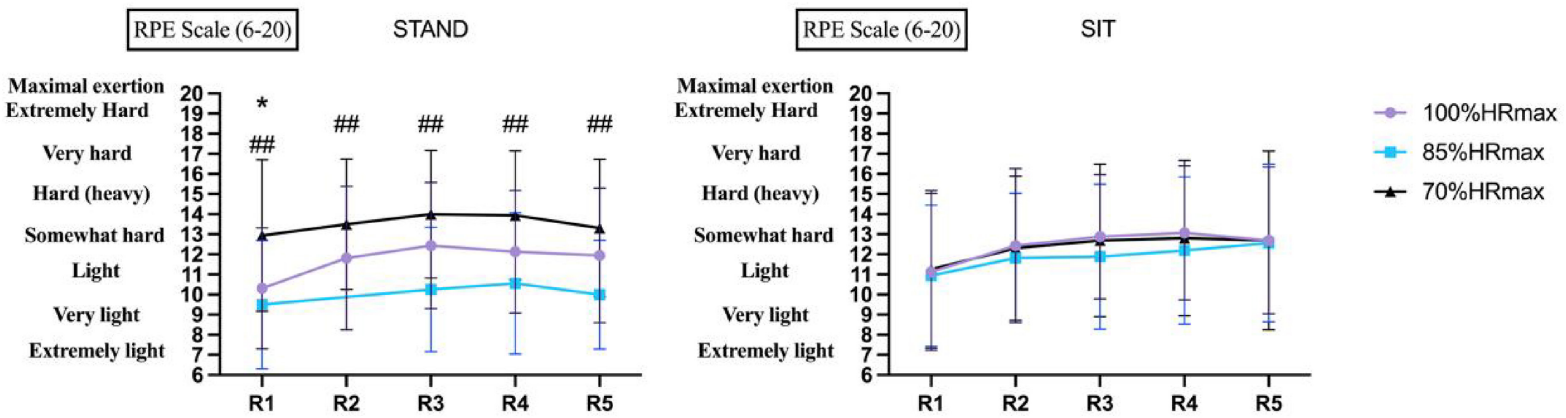
Effect of varied load intensities on rate of perceived exertion (RPE). *Significant difference compared with 100% HRmax. ^##^Significant difference compared with 85% HRmax.

As shown in Figure 9, in transition round 1, rating of perceived exertion (RPE) was significantly different in 70% HRmax relative to 100% HRmax (F = 0.131, *t* = −2.563, *p* = 0.04) and was significantly higher in 70% HRmax than in 85% HRmax in conversion round 1 (F = 0.599, *t* = −3.076, *p* < 0.05); in conversion rounds 3 (F = 0.214, *t* = −2.290, *p* < 0.05) and R4 (F = 0.321, *t* = −2.127, *p* < 0.05), 70% HRmax’s subjective physical sensation was significantly higher than 85% HRmax’s.

## Discussion

### The effect of varied load intensities on the number of shots hit

Previous studies have demonstrated that increased heart rate (HR) during intense exercise is closely associated with greater physiological tremor and reduced postural stability, both of which directly impair shooting accuracy in biathlon. Elevated HR reflects heightened cardiovascular strain and sympathetic activation, which can increase muscle tremor, reduce balance control, and disrupt fine motor control during aiming. As a result, shooting performance declines with increasing exercise intensity and higher HR. These relationships are well-documented in the literature examining the psychophysiological determinants of shooting performance, highlighting the critical role of HR control, postural stability, and tremor reduction in achieving high shooting accuracy under biathlon-specific conditions.

Shooting plays a crucial role in the performance of biathletes during competition. The winner of two silver and one bronze medals at the Sochi Winter Olympics, Arnd Peiffer (2024), once said: “My shooting is the absolute key to success in the competition, with a hit rate of maybe 96%.” This quote highlights the importance of high hit rates in biathlon competitions and underscores the impact shooting precision can have on an athlete’s overall performance and chances of success.

In terms of shooting hits, the number of total hits is the main indicator of an athlete’s shooting consistency, in addition to shooting time. The results revealed that the total number of hits was significantly higher for 85% HRmax intervals compared to no intervals, the total number of hits was significantly lower for 70% HRmax intervals compared to 85% HRmax intervals. Additionally, the total hit rate was significantly lower for 70% HRmax intervals than for 85% HRmax intervals (Ohtonen et al., 2020). The study also examined the firing efficiency by calculating the ratio of the total number of hits to the total firing time. The results revealed that the efficiency value of the 85% HRmax interval was superior to both the 70% HRmax interval and the no-interval condition, indicating that the 85% HRmax interval maximized shooting accuracy stability. This suggests that, in terms of maintaining shooting stability, the 85% HRmax interval is the most effective in optimizing shooting accuracy (Vine et al., 2014). No interval and interval of 70% HRmax are not conducive to maintaining shooting stability (Botvinick and Braver, 2015); from the shooting stability maintenance perspective, too long a transition time tends to reduce the total number of hits of the athlete.

### The effect of varied load intensities on shooting time before shooting

First, in terms of shooting stability, the first shot time indicator is seen by scholars from various countries as a key indicator for evaluating shooting stability in the biathlon transition segment. It is related to the previous skating segment and directly affects the athlete’s residence time in the shooting area (Sattlecker et al., 2015). Therefore, the first shot time index is an important indicator reflecting the athletes’ skating physical reserve and shooting stability. In this sense, athletes must grasp the first shot time in each transition round to improve their hit rate of shooting (Leirdal et al., 2013).

The results of the study showed that in the R3 and R5 conversion rounds, the first shot time with 85% HRmax was significantly lower than that without interval, and the first shot time with 70% HRmax was not significantly different from that without interval. In the R3 and R5 conversion rounds, the first shot time with a 70% HRmax was significantly higher than the first shot time with an 85% HRmax. In terms of this result, the further back in the transition rounds in biathlon, the faster the athlete demonstrated a faster first shot time after an 85% HRmax (Schillinger et al., 2013; Sattlecker et al., 2013). This suggests that for short-distance events, different transition times did not show a significant effect on the first shot time, but with increasing exercise time and increasing number of transition rounds, for long-distance events, either too short or too long transition session time was detrimental to the first shot time, and the 85% HRmax presented a shorter firing time (Cooke, 2013). The longer shooting time observed at 70% HRmax was somewhat unexpected, as lower exercise intensity is typically associated with greater stability. However, this finding may be explained by differences in arousal level and task engagement. At 85% and 100% HRmax, athletes maintained higher sympathetic activation and task focus, enabling faster aiming and trigger release. In contrast, the lower intensity at 70% HRmax may have led to reduced arousal, decreased motor readiness, and longer aiming times as athletes adopted a more cautious shooting strategy. This suggests that an optimal arousal level exists for rapid, accurate shooting in biathlon, and excessively low intensity may not necessarily improve temporal performance.

This shows that the awareness of increasing the shooting speed has its necessary, and the skiing time can be extended based on fast speed, which helps to improve the overall performance. In a research review published by Swedish scholars Marko S. Laaksonen and H-C Holmberg, Austrian scholar Thomas Finkenzeller, and Norwegian sports scientist Gerold Sattlecker, it was mentioned that “over the course of a season, world-class biathletes fire over 20,000 shots in more than 200 training sessions, training focuses on improving accuracy and speed in preparation, firing and exiting the shooting zone. A total of 10 Many world-class biathletes focus specifically on preparing for the first shot quickly and exiting the firing area as quickly as possible.” As you can see, the speed of the first shot is the key to the trend of the competitive ability of the best international biathletes, while completing the shooting procedure and leaving the shooting area as quickly as possible is often reflected in the athletes’ last shot performance (Hoffman et al., 2013; Konttinen and Lyytinen, 1992; Hoffman and Street, 1992).

The final shot time is a significant indicator of an athlete’s shooting stability in actual biathlon competitions. Although most studies by scholars from various countries primarily focus on the first shot time in each conversion round, Köykkä is one of the few who mentioned the importance of the end shot time index in a review of related studies. It is worth noting that different distance events in biathlon require varying shooting performance, as supported by evidence from numerous studies. However, there is currently a lack of research specifically examining the time-to-end shot index. Further studies in this area could provide valuable insights into optimizing shooting performance across different biathlon events (Köykkä et al., 2020). Therefore, the present study attempted to investigate the relationship between end shot time and each transition round. The results showed that for each conversion round, the final firing time with a 70% HRmax was significantly higher than the final firing time with an 85% HRmax in the 2nd conversion round, while no significant differences were observed in other conversion rounds, indicating that for short distances, the longer the interval, the longer the final firing time, while in long distances, the final firing time was not influenced by the interval.

### Analysis of the effects of sitting and standing posture on physiological and psychological responses and shooting stability

The recovery ability of the organism is closely related to energy metabolism, and the aerobic energy supply system is worthy of attention. In biathlon, athletes need to complete high-intensity cross-country skiing alternating with shooting events (Tervo and Jensen, 2010; Stoggl et al., 2010). The rapid recovery of physiological functions after each round of skiing affects shooting stability to some extent, and the transition session requires rapid and steady decrease in HR, and the ability to rapidly regulate the respiratory system (Alsobrook and Heil, 2009). Some studies have shown that while some biathletes perform well in the first half of the opening round, they are unable to maintain their performance level for the rest of the competition. During the conversion session, the aerobic energy supply system is important for the relief and elimination of fatigue. In recent years, several studies have begun to focus on the effects of different recovery modes on repetitive high-intensity exercise in cross-country skiing, and also gradually focused their research on the rapid recovery of energy metabolic factors on the nervous system, cardiovascular system, and respiratory system within the organism during repeated intense gliding (Vanlandewijck, 2006). Some researchers have explored the effect of energy metabolism on athletic performance from the perspective of switching rounds, and Manfredini et al. (2002) found that a high aerobic capacity reduced athlete fatigue during four rounds, which not only improved athletic performance in each round but also promoted consistent performance between rounds. In biathlon, Tønnessen et al. (2015) concluded that good shooting stability can compensate to some extent for the lower aerobic capacity of athletes. Therefore, energy metabolism plays an important connecting role in the maintenance of shooting stability in the conversion link (Tønnessen et al., 2015; Rapp et al., 2013).

For the use of the double pole technique, the standing posture requires the participation of skeletal muscles throughout the athlete’s body, and with the mobilization of a greater proportion of nerves and muscles involved, a portion of the blood supply from the heart to the upper and lower extremities will be transported to the muscles of the lower extremities involved in the exercise, and with the prolongation of the exercise time, the proportion of the glycolytic energy supply system and the aerobic oxidative energy supply system involved in energy supply increases under moderate load intensity exercise conditions, causing the level in the body increased (Leirdal et al., 2013; Hoffman and Clifford, 1990; Vergès et al., 2003). In terms of transition time, there was no significant difference between the three groups in round one. However, from rounds 2 to 5, blood lactate concentration was significantly higher in the 70% HRmax group than in the 85% HRmax group. In addition, by the fifth round, blood lactate concentration in the intermittent 1-min group had gradually decreased close to that of the first round. In the temporal dimension, the trend of blood lactate concentration in the no-interval group and the 70% HRmax group was rising, then falling, then rising again, while the trend of blood lactate concentration in the 85% HRmax group was rising and then falling (Manfredini et al., 2002; Patton and Duggan, 1987; Undebakke et al., 2019).

In terms of RPE during shooting, the cognitive ability required for perceptual assessment of shooting stability is not affected by strenuous skiing and is not associated with the observed decrease in shooting stability after strenuous exercise (Laaksonen et al., 2020; Laaksonen et al., 2018a; Jonsson Kårström et al., 2019). Good biathletes can accurately assess their shooting performance under both quiet and loaded conditions. However, strenuous exercise increases the difficulty of motor perception assessment and short-term memory of shooting performance. The RPE at shooting index represents the change in the subjective physical strength perception of the athlete during the transition from skiing to shooting, a change that is also a unique competitive characteristic of biathlon, where the body perceives its physical strength differently during the transition from motion to rest. It has been demonstrated that the mean value of RPE during the shooting of athletes can be used to analyze the steady-state situation during this phase, and from an overall perspective, RPE was significantly correlated with biathlon performance and total skiing time (*r* = 0.64–0.95, *P* < 0.05). Thus, RPE at the time of shooting was also related to competition performance to some extent (Hoffman et al., 2013; Luchsinger et al., 2019b; Sattlecker and Rampl, 2010; Ihalainen et al., 2018).

Swedish physiologist Gunnell Borg developed the Rating of Subjective Physical Effort (RPE) scale to determine the degree of fatigue based on the rating of self-perception indicated by exercisers during exercise, allowing semi-quantitative analysis of an otherwise crude qualitative analysis. Research on the subjective physical perception scale in biathlon began with a paper by Joan N. Vickers from the University of Calgary published in the Journal of Motor Behavior in 2007, which showed that the RPE was able to reflect the subjective physical perception of biathletes at different exercise intensities, and the results were significant as the exercise significantly different levels of intensity [F(4,36) = 172.58, *p* < 0.0001], but there was no interaction between stress and exercise intensity (Vickers and Williams, 2007). This suggests that in biathlon, RPE is a test index that is controlled only by exercise intensity and not by stress. When exercising at exercise intensity (55%, 70%, 85%, and 100%) of maximum HR, HR, subjective physical sensation (RPE), and quiet eye time (QE) were key predictors when the load intensity was 55% (F = 5.83, *p* = 0.03 < 0.05, *r* = 0.86) and when the load intensity was 85%, subjective physical sensation (RPE) was (F = 6.14, *p* = 0.04 < 0.05, *r* = 0.66), when the load intensity was 100%, subjective physical perception (RPE) and quiet eye time (QE) were the key predictors (F = 7.31, *p* = 0.02 < 0.05, *r* = 0.82) (Sattlecker et al., 2014; Baca and Kornfeind, 2012; Larue et al., 1989; Konttinen et al., 2010; Kerick et al., 2001). Thus, the key predictor corresponding to the load intensity of 85% of the maximum HR in this study. This also provides a theoretical basis for the selection of this index in this study (Ihalainen et al., 2018; Vine et al., 2014; Björklund, 2018).

## Limitations

Due to the training and competition cycle of national team athletes, there are only 16 male para-biathlon athletes who have time to participate in the test. Although the sample size is small, the selected subjects have sufficient exercise levels and have certain reference value. In the future, there is an opportunity to expand the sample size of the test and increase the comparison of different gender athletes. In addition, the temperature control in this experiment may not be the same as in the outdoor environment as in the biathlon sports environment (Ihalainen et al., 2018; Luchsinger et al., 2016; Tweedy and Vanlandewijck, 2010).

## Conclusion

The load intensity of 85% HRmax has the best effect on para-biathlon athletes. Under this intensity, athletes have the shortest shooting time, the largest number of total shooting hits, and the highest hit rate and transition efficiency.

For the shooting performance after standing slide and sitting slide, keeping the load intensity of 100% HRmax will affect the athlete’s first shooting time, and keeping the load intensity of 70% HRmax will affect the athlete’s conversion efficiency.

In terms of physiological and psychological performance, standing gliding with different load intensity has an obvious influence on BLA and RPE, but sitting gliding with different load intensity has no effect on BLA and RPE.

### The practical applications

To optimize para-biathlon shooting performance, coaches should guide athletes to maintain an optimal heart rate of 85% HRmax during the skiing-to-shooting transition—this intensity balances stability, arousal, and motor readiness, outperforming both 100% (excessive tremor) and 70% (reduced readiness) HRmax. Brief recovery strategies (e.g., controlled breathing) can lower HR from high-intensity levels to 85% HRmax, while low-intensity activation drills address deficits at 70% HRmax; posture-specific training is also critical, with standing drills focusing on core/limb stability and seated drills prioritizing fine motor control of aiming and trigger release.

Coaches should use the study’s efficiency performance (EP) metric to balance athletes’ shooting speed and accuracy, integrating simulated competition drills with immediate feedback to refine strategies. Additionally, training athletes to self-monitor exertion via the 6–20 Borg RPE scale (targeting 13–15, corresponding to 85% HRmax) and reviewing RPE-HR data post-training enables personalized intensity adjustment; practicing with standard IBU targets and visual cue training further enhances shooting efficiency at optimal intensity, translating study findings into improved competition outcomes.

## Data Availability

The raw data supporting the conclusions of this article will be made available by the authors, without undue reservation.

## References

[B1] Alsobrook N. G. Heil D. P. (2009). Upper body power as a determinant of classical cross-country ski performance. *Eur. J. Appl. Physiol*. 105 633–641. 10.1007/s00421-008-0943-z 19039602

[B2] Baca A. Kornfeind P. (2012). Stability analysis of motion patterns in biathlon shooting. *Hum. Mov. Sci.* 31 295–302. 10.1016/j.humov.2010.05.008 20675002

[B3] Benum S. D. van der Weel F. R. van der Meer A. L. H. (2021). In a heartbeat: Prospective control of cardiac responses for upcoming action demands during biathlon. *Ecol. Psychol.* 2 90–95. 10.1080/10407413.2021.1885979

[B4] Björklund G. (2018). Shooting efficiency for winners of World Cup and World Championship races in men’s and women’s biathlon: Where is the cut-off? *Int. J. Perform. Anal. Sport* 18 545–553. 10.1080/24748668.2018.1497920

[B5] Botvinick M. Braver T. (2015). Motivation and cognitive control: From behavior to neural mechanism. *Annu. Rev. Psychol*. 66 83–113. 10.1146/annurev-psych-010814-015044 25251491

[B6] Cooke A. (2013). Readying the head and steadying the heart: A review of cortical and cardiac studies of preparation for action in sport. *Int. Rev. Sport Exerc. Psychol.* 6 122–138. 10.1080/1750984X.2012.724438

[B7] Gallicchio G. Finkenzeller T. Sattlecker G. Lindinger S. Hoedlmoser K. (2019). The influence of physical exercise on the relation between the phase of cardiac cycle and shooting accuracy in biathlon. *Eur. J. Sport Sci*. 19 567–575. 10.1080/17461391.2018.1535626 30362887 PMC6518456

[B8] Gallicchio G. Finkenzeller T. Sattlecker G. Lindinger S. Hoedlmoser K. (2016). Shooting under cardiovascular load: Electroencephalographic activity in preparation for biathlon shooting. *Int. J. Psychophysiol*. 109 92–99. 10.1016/j.ijpsycho.2016.09.004 27619492

[B9] Hakkarainen A. Linnamo V. Lindinger S. (2016). *Science and Nordic Skiing III.* Jyväskylä: University of Jyväskylä.

[B10] Heinrich A. Hansen D. W. Stoll O. Cañal-Bruland R. (2020). The impact of physiological fatigue and gaze behavior on shooting performance in expert biathletes. *J. Sci. Med. Sport* 23 883–890. 10.1016/j.jsams.2020.02.010 32146083

[B11] Heinrich A. Stoll O. Cañal-Bruland R. A. (2021). Biopsychosocial framework to guide interdisciplinary research on biathlon performance. *Front. Psychol*. 12:671901. 10.3389/fpsyg.2021.671901 33995230 PMC8116492

[B12] Hoffman M. D. Clifford P. S. (1990). Physiological responses to different cross country skiing techniques on level terrain. *Med. Sci. Sports Exerc*. 22 841–842. 10.1249/00005768-199012000-00017 2287263

[B13] Hoffman M. D. Street G. M. (1992). Characterization of the HR response during biathlon. *Int. J. Sports Med.* 13 390–394. 10.1055/s-2007-1021286 1521956

[B14] Hoffman M. D. Gilson P. M. Westenburg T. M. Spencer W. A. (2013). Biathlon shooting performance after exercise of different intensities. *Int. J. Sports Med*. 13 270–273. 10.1055/s-2007-1021265 1601564

[B15] Ihalainen S. Laaksonen M. S. Kuitunen S. Leppävuori A. Mikkola J. Lindinger S. J. et al. (2018). Technical determinants of biathlon standing shooting performance before and after race simulation. *Scand. J. Med. Sci. Sports* 28 1700–1707. 10.1111/sms.13072 29446507

[B16] Jonsson Karstrom M. McGawley K. Laaksonen M. S. (2019). Physiological responses to rifle carriage during roller-skiing in elite biathletes. *Front. Physiol*. 10:1519. 10.3389/fphys.2019.01519 31956312 PMC6951403

[B17] Jonsson Kårström M. McGawley K. Laaksonen M. S. (2019). Physiological responses to rifle carriage during roller-skiing in elite biathletes. *Front. Physiol*. 10:1519. 10.3389/fphys.2019.01519 31956312 PMC6951403

[B18] Kerick S. E. McDowell K. Hung T. M. Santa Maria D. L. Spalding T. W. Hatfield B. D. (2001). The role of the left temporal region under the cognitive motor demands of shooting in skilled marksmen. *Biol. Psychol*. 58 263–277. 10.1016/s0301-0511(01)00116-8 11698117

[B19] Konttinen N. Lyytinen H. (1992). Physiology of preparation: Brain slow waves, HR, and respiration preceding triggering in rifle shooting. *Int. J. Sport Psychol.* 23 110–127.

[B20] Konttinen N. Lyytinen H. Viitasalo J. (2010). Rifle-balancing in precision shooting: Behavioral aspects and psychophysiological implication. *Scand. J. Med. Sci. Sports* 8 78–83. 10.1111/j.1600-0838.1998.tb00172.x 9564711

[B21] Köykkä M. Ihalainen S. Linnamo V. Ruotsalainen K. Häkkinen K. Laaksonen M. S. (2020). Aiming strategy affects performance-related factors in biathlon standing shooting. *Scand. J. Med. Sci. Sports* 31 573–585. 10.1111/sms.13864 33113219

[B22] Laaksonen M. S. Andersson E. Jonsson Kårström M. Lindblom H. McGawley K. (2020). Laboratory-based factors predicting skiing performance in female and male biathletes. *Front. Sports Act. Living* 2:99. 10.3389/fspor.2020.00099 33345089 PMC7739653

[B23] Laaksonen M. S. Finkenzeller T. Holmberg H. C. Sattlecker G. (2018a). The influence of physiobiomechanical parameters, technical aspects of shooting, and psychophysiological factors on biathlon performance: A review. *J. Sport Health Sci*. 7 394–404. 10.1016/j.jshs.2018.09.003 30450247 PMC6234024

[B24] Laaksonen M. S. Jonsson M. Holmberg H. C. (2018b). The olympic biathlon - recent advances and perspectives after pyeongchang. *Front. Physiol*. 9:796. 10.3389/fphys.2018.00796 30013486 PMC6036135

[B25] Larue J. Bard C. Otis L. Fleury M. (1989). [Stability in shooting: The effect of expertise in the biathlon and in rifle shooting]. *Can. J. Sport Sci*. 14 38–45. 2924221

[B26] Leirdal S. Sandbakk O. Ettema G. (2013). Effects of frequency on gross efficiency and performance in roller ski skating. *Scand. J. Med. Sci. Sports* 23 295–302. 10.1111/j.1600-0838.2011.01379.x 22092985

[B27] Luchsinger H. Kocbach J. Ettema G. Sandbakk Ø. (2020). Contribution from cross-country skiing, start time and shooting components to the overall and isolated biathlon pursuit race performance. *PLoS One* 15:e0239057. 10.1371/journal.pone.0239057 32925963 PMC7489554

[B28] Luchsinger H. Kocbach J. Ettema G. Sandbakk O. (2019a). The Contribution From Cross-Country Skiing and Shooting Variables on Performance-Level and Sex Differences in Biathlon World Cup Individual Races. *Int J Sports Physiol Perform*. 14 190–195. 10.1123/ijspp.2018-0134 30039989

[B29] Luchsinger H. Sandbakk Ø Schubert M. Ettema G. Baumeister J. (2016). A comparison of frontal theta activity during shooting among biathletes and cross-country skiers before and after vigorous exercise. *PLoS One* 11:e0150461. 10.1371/journal.pone.0150461 26981639 PMC4794229

[B30] Luchsinger H. Talsnes R. K. Kocbach J. Sandbakk Ø. (2019b). Analysis of a biathlon sprint competition and associated laboratory determinants of performance. *Front. Sports Act. Living* 1:60. 10.3389/fspor.2019.00060 33344983 PMC7739577

[B31] Maier T. Meister D. Trösch S. Wehrlin J. P. (2019). Predicting biathlon shooting performance using machine learning. *J. Sports Sci*. 36 2333–2339. 10.1080/02640414.2018.1455261 29565223

[B32] Manfredini F. Manfredini R. Carrabre J. E. Litmanen H. Zhukovskaja L. Dal Follo D. et al. (2002). Competition load and stress in sports: A preliminary study in biathlon. *Int. J. Sports Med*. 23 348–352. 10.1055/s-2002-33140 12165886

[B33] Ohtonen O. Linnamo V. Göpfert C. (2020). “Effect of 20 km simulated race load on propulsive forces during ski skating science and skiing VIII,” in *Science and Skiing VIII : 8th International Congress on Science and Skiing*, eds Karczewska-Lindinger M. Hakkarainen A. Linnamo V. Lindinger S. (Jyväskylä: University of Jyväskylä), 130–137.

[B34] Ortega E. Wang C. J. K. (2018). Pre-performance physiological state: Heart rate variability as a predictor of shooting performance. *Appl. Psychophysiol. Biofeedback* 43 75–85. 10.1007/s10484-017-9386-9 29124507

[B35] Panissa V. L. Cal Abad C. C. Julio U. F. Andreato L. V. Franchini E. (2016). High-intensity intermittent exercise and its effects on heart rate variability and subsequent strength performance. *Front. Physiol*. 7:81. 10.3389/fphys.2016.00081 26973543 PMC4777986

[B36] Patton J. F. Duggan A. (1987). Upper and lower body anaerobic power: Comparison between biathletes and control subjects. *Int. J. Sports Med*. 8 94–98. 10.1055/s-2008-1025648 3596883

[B37] Peiffer A. (2024). *Journal of Biathlon Coaching*. Biathlon Union (IBU) Academy.

[B38] Pelin B. I. Mereuta C. (2018). Improvement of shooting technical skills in the shooting range within the biathlon test for juniors. *Series IX* 11 179–184.

[B39] Rapp W. Lappi T. Lindinger S. Sattlecker G. Schillinger W. Hakkarainen A. et al. (2013). “Neuromuscular Control and Cognitive Processes in Skiing Performance”, *Paper presented at the 6th International Congress on Science & Skiing*, St. Moritz.

[B40] Rosso V. Rapp W. Sattlecker G. Birklbauer J. Rampl J. Schillinger W. et al. (2017). “Robotic assistance in winter sports: Applications and future perspectives,” Paper presented at the *International Conference on Robotics in Alpe-Adria Danube Region*, Graz.

[B41] Sattlecker G. Birklbauer J. Rapp W. Lappi T. Lindinger S. Schillinger W. et al. (2013). “Biomechanical analysis of skiing movements: Implications for performance enhancement,” *Paper presented at the 6th International Congress on Science & Skiing*, St. Moritz.

[B42] Sattlecker G. Buchecker M. Rapp W. Lappi T. Lindinger S. Sattlecker L. S. et al. (2015). “Physiological adaptations to nordic skiing: A multidisciplinary approach,” *Paper presented at the 3rd International Congress on Science and Nordic Skiing*, Trondheim.

[B43] Sattlecker G. Buchecker M. Gressenbauer C. Müller E. Lindinger S. J. (2017). Factors discriminating high from low score performance in biathlon shooting. *Int. J. Sports Physiol. Perform*. 12 377–384. 10.1123/ijspp.2016-0195 27348149

[B44] Sattlecker G. Buchecker M. Müller E. Lindinger S. J. (2014). Postural balance and rifle stability during standing shooting on an indoor gun range without physical stress in different groups of biathletes. *Int. J. Sports Sci. Coach.* 9 171–183. 10.1260/1747-9541.9.1.171

[B45] Sattlecker L. S. Rampl J. (2010). “Technological and nutritional interventions to improve sports performance and wellbeing,” *Paper presented at the Multidisciplinary Research Symposium: Sports and Wellbeing–Technological and Nutritional Aspects*, Vienna.

[B46] Schillinger W. Hakkarainen A. Linnamo V. Lindinger S. (2013). “Cognitive factors influencing skiing expertise: attention and decision-making,” *Paper presented at the International Congress on Science and Skiing*, St. Moritz.

[B47] Skiing W. P. N. (2019). Rules and Regulations 2019/2020. *World Para Nord. Skiing* 11 100–105.

[B48] Stoggl T. Enqvist J. Muller E. Holmberg H. C. (2010). Relationships between body composition, body dimensions, and peak speed in cross-country sprint skiing. *J. Sports Sci*. 28 161–169. 10.1080/02640410903414160 20391090

[B49] Stöggl T. Lindinger S. Müller E. (2016). Analysis of a simulated sprint competition in classical cross country skiing. *Scand. J. Med. Sci. Sports* 17 362–372. 10.1111/j.1600-0838.2006.00589.x 16911588

[B50] Tervo J. Jensen R. (2010). “Biomechanical patterns in elite and recreational sports performance,” *Paper presented at the 28th International Conference on Biomechanics in Sports*, Auckland.

[B51] Tønnessen E. Haugen T. A. Hem E. Leirstein S. Seiler S. (2015). Maximal aerobic capacity in the winter-Olympics endurance disciplines: Olympic-medal benchmarks for the time period 1990-2013. *Int. J. Sports Physiol. Perform*. 10 835–839. 10.1123/ijspp.2014-0431 25611016

[B52] Tweedy S. M. Vanlandewijck Y. C. (2010). International Paralympic Committee position stand–background and scientific principles of classification in Paralympic sport. *Br. J. Sports Med*. 45 259–261. 10.1136/bjsm.2009.065060 19850575

[B53] Undebakke V. Berg J. Tjønna A. E. Sandbakk Ø. (2019). Comparison of physiological and perceptual responses to upper-, lower-, and whole-body exercise in elite Cross-Country Skiers. *J. Strength Cond. Res*. 33 1086–1094. 10.1519/JSC.0000000000003078 30741871

[B54] Vanlandewijck Y. (2006). Sport science in the paralympic movement. *J. Rehabil. Res. Dev*. 43 xvii–xxiv. 10.1682/jrrd.2006.07.0078 17436167

[B55] Vergès S. Flore P. Favre-Juvin A. (2003). Blood lactate concentration/HR relationship: Laboratory running test vs. field roller skiing test. *Int. J. Sports Med.* 24 446–451. 10.1055/s-2003-41176 12905094

[B56] Vickers J. N. Williams A. M. (2007). Performing under pressure: The effects of physiological arousal, cognitive anxiety, and gaze control in biathlon. *J. Mot. Behav*. 39 381–394. 10.3200/JMBR.39.5.381-394 17827115

[B57] Vine S. J. Moore L. J. Wilson M. R. (2014). Quiet eye training: The acquisition, refinement and resilient performance of targeting skills. *Eur. J. Sport Sci*. 14 (Suppl. 1), S235–S242. 10.1080/17461391.2012.683815 24444212

